# Community Attitudes to the Appropriation of Mobile Phones for Monitoring and Managing Depression, Anxiety, and Stress

**DOI:** 10.2196/jmir.1475

**Published:** 2010-12-19

**Authors:** Judith Proudfoot, Gordon Parker, Dusan Hadzi Pavlovic, Vijaya Manicavasagar, Einat Adler, Alexis Whitton

**Affiliations:** ^2^Black Dog InstituteSydneyAustralia; ^1^School of PsychiatryUniversity of New South WalesSydneyAustralia

**Keywords:** Mobile phones, monitoring, self-help, depression, anxiety, stress, Internet intervention

## Abstract

**Background:**

The benefits of self-monitoring on symptom severity, coping, and quality of life have been amply demonstrated. However, paper and pencil self-monitoring can be cumbersome and subject to biases associated with retrospective recall, while computer-based monitoring can be inconvenient in that it relies on users being at their computer at scheduled monitoring times. As a result, nonadherence in self-monitoring is common. Mobile phones offer an alternative. Their take-up has reached saturation point in most developed countries and is increasing in developing countries; they are carried on the person, they are usually turned on, and functionality is continually improving. Currently, however, public conceptions of mobile phones focus on their use as tools for communication and social identity. Community attitudes toward using mobile phones for mental health monitoring and self-management are not known.

**Objective:**

The objective was to explore community attitudes toward the appropriation of mobile phones for mental health monitoring and management.

**Methods:**

We held community consultations in Australia consisting of an online survey (n = 525), focus group discussions (n = 47), and interviews (n = 20).

**Results:**

Respondents used their mobile phones daily and predominantly for communication purposes. Of those who completed the online survey, the majority (399/525 or 76%) reported that they would be interested in using their mobile phone for mental health monitoring and self-management if the service were free. Of the 455 participants who owned a mobile phone or PDA, there were no significant differences between those who expressed interest in the use of mobile phones for this purpose and those who did not by gender (χ2_1_, = 0.98, *P* = .32, phi = .05), age group (χ2_4_, = 1.95, *P* = .75, phi = .06), employment status (χ2_2_, = 2.74, *P* = .25, phi = .08) or marital status (χ2_4_, = 4.62, *P* = .33, phi = .10). However, the presence of current symptoms of depression, anxiety, or stress affected interest in such a program in that those with symptoms were more interested (χ^2^_1_, = 16.67, *P* < .001, phi = .19). Reasons given for interest in using a mobile phone program were that it would be convenient, counteract isolation, and help identify triggers to mood states. Reasons given for *lack* of interest included not liking to use a mobile phone or technology, concerns that it would be too intrusive or that privacy would be lacking, and not seeing the need. Design features considered to be key by participants were enhanced privacy and security functions including user name and password, ease of use, the provision of reminders, and the availability of clear feedback.

**Conclusions:**

Community attitudes toward the appropriation of mobile phones for the monitoring and self-management of depression, anxiety, and stress appear to be positive as long as privacy and security provisions are assured, the program is intuitive and easy to use, and the feedback is clear.

## Introduction

Reducing the burden of mental disease requires a combination of effective prevention, early intervention, treatment, and self-management, and a critical aspect of these functions is for individuals to monitor their mental health. Self-monitoring brings about actual improvements in mood and behavior and enhances individuals’ compliance with treatments [1,2].

Historically, paper diaries have been the primary mode of monitoring, but patients can find them cumbersome. Noncompliance is also common, as demonstrated by Stone et al [3] who compared patients’ actual and reported compliance with diary keeping. By embedding a photosensor into the binder containing the paper diary forms that detected light and recorded when the binder was opened and closed, the researchers ascertained that actual compliance was 11%, whereas participant-reported compliance was 90%. In contrast, compliance with an electronic diary was 94% [3]. However, computer-based monitoring also has inherent limitations in that it relies on users being at their computer at scheduled times, which can be inconvenient, and, as a result, nonadherence after short periods of time is also common with this delivery channel. Retrospective recall of symptoms, mood, or behavior can also be unreliable [3]. To be maximally effective, individual self-monitoring needs to take place regularly and in real time to reduce recall bias and increase accuracy.

Mobile phones offer a solution. Their take-up has reached saturation point in most developed countries and is increasing in developing countries; they are carried on the person and they are usually turned on. A further advantage is that mobile phones allow the gathering of frequent instantaneous reports of mood and behavior while people go about their everyday business. Termed Ecological Momentary Assessment, or EMA, mobile phone monitoring has been shown to more accurately represent the true natural history of transitory states than dispersed measurements and may decrease user burden [4]. With mobile phones, users can be prompted to respond, and these “just-in-time” prompts can be scheduled for key times. Mobile phone monitoring is also potentially more convenient than paper- or computer-based recording.

Mobile phones have been used for monitoring within behavioral health applications such as alcohol consumption [5] and gambling [6]. They have also been used to deliver simple interventions, for example, to manage migraines [7], enhance physical activity [8], cease smoking [9], and control weight [10]. However, the use of mobile phones in mental health monitoring and management is in its infancy.

Furthermore, community attitudes toward the use of mobile phones as a mental health tool are unknown. Until recently, mobile phones were primarily viewed as tools of communication and social identity, but increasingly they are evolving into a personal multipurpose tool for their owners, with routine functions now including reminders for medical appointments, timekeeping, and note taking. While preliminary research has been undertaken exploring community attitudes toward the use of mobile phones for health monitoring, such as for asthma [11], attitudes to the appropriation of mobile phones for mental health monitoring and management have not yet been investigated.

Derived from the field of marketing, the term “appropriation” refers to the processes that take place when new uses are invented for existing technologies and when these uses develop into routine practices and spread within a user community [12]. The aim of this study was to investigate community attitudes toward the appropriation of mobile phones for mental health monitoring and management as groundwork for the development of a digital tool for self-monitoring and self-management of depression, anxiety, and stress.

## Methods

A mixed method approach was used consisting of an online survey, focus group discussions, and interviews. The target population was Australian adults over 18 years of age with or without depression, anxiety, or stress. Participation was voluntary and anonymous in all 3 study components.

### Development and Administration of Study Tools

Questions for the online survey, focus group discussions, and interviews were generated, reviewed, and amended by the research group in consultation with the members of the community program team at the Black Dog Institute, a mood disorders unit in Australia. The final set of questions explored the following issues:

- current usage of mobile phones
- attitudes toward using a program on mobile phones or the Internet for monitoring and managing depression, anxiety, or stress
- possible ways in which participants might use such a program
- any key features that such a program should have
- demographic information and mental health history of participants

The survey was pilot tested using QuestionPro [13]. The survey’s usability and functionality were assessed and improvements made prior to it being posted on the website of the Black Dog Institute [14]. The website provides information and tools about mood disorders for the public, health professionals, and workplaces. A broad cross section of the public uses the site, including those with mood disorders and those without (eg family, carers, friends, students, and interested individuals). The site is consistently in the top 5 Google ranks for mood disorders in Australia.

The survey was voluntary and was open to any site visitor. Initial contact with potential participants was made on the Internet. The survey consisted of 46 questions over 12 Web pages. Some pages had more items than others, although the maximum number of items per page did not exceed 7. Forty-four of the 46 items were mandatory, each was highlighted, and it was not possible for respondents to proceed to the next page until they had completed the mandatory items. Because there was no back button, respondents were unable to review or change their answers. To prevent multiple entries, a cookie was placed on the participant’s computer. There was no Internet protocol (IP) check or log file analysis. As this was an open survey, no log-in or registration was required. The data were collected over a 3-month period from May to August 2009.

Focus group participants and interviewees were recruited through community organizations, a variety of companies, and the Black Dog Institute community programs. Similar questions to those in the online survey were used in the focus group discussions and the interviews to facilitate later triangulation of results. To protect participants’ confidentiality within the interviews and focus groups, demographic information and mental health history were completed anonymously by a paper and pencil questionnaire that participants sealed in an envelope and gave to the researcher. An experienced moderator conducted each of the focus groups with 1 or 2 observers present who took notes. The sessions were audio taped for later transcription and analysis.

### Informed Consent Process

Individuals who clicked on the link to the online survey were provided with a statement about the study that included its purpose, the length of time to complete the survey (approximately 15 minutes), how the data were being stored (initially on the secure QuestionPro server then in password-protected files in the university’s secure server for 7 years), and the name of the chief investigator (author JP), before being invited to give their informed consent online and to access the survey. The survey was anonymous. However, those who wished to enter a draw to win an iPod Nano for completing the survey were invited to separately provide their name, phone number, and email address. This information was stored in a secure password-protected file on the QuestionPro server and then transferred to the university’s secure server for long-term storage.

The informed consent process for the focus group discussions and interviews was similar. Individuals who expressed interest in being interviewed or participating in the focus group discussions were provided with a written outline of the study which included its purpose, the length of time the interview/focus group would take (approximately one hour), how the data were being stored (audiotaped for later transcription and storage for 7 years in password-protected files in the university’s secure server) and the name of the chief investigator (author JP). They were then invited to give their informed consent to participate in the interview or focus group. No identifying information was collected.

The study was approved by the University of New South Wales Human Research Ethics Committee.

### Advertising and Recruitment

The online survey, focus group discussions, and interviews were advertised simultaneously through Facebook, the websites of the University of New South Wales and Black Dog Institute, and the intranets of a variety of companies and consumer organizations. The advertisement for the online survey can be found in Multimedia Appendix 1. The advertisements for the interviews and focus groups had similar wording to the advertisement in Multimedia Appendix 1 with the exception that participants were reimbursed A$50 for their time and travel expenses instead of being given the opportunity to enter into the draw for the iPod Nano.

### Participants

From May to August 2009, 655 unique visitors accessed the online survey of whom 48 were ineligible because they were either under 18 years old (n=13) or did not live in Australia (n=35). Of the 607 eligible respondents, 525 (86.5%) completed the survey.

In all, 6 focus group discussions involving 47 participants (70% female) were conducted from June to August 2009; 4 groups were held in urban areas and 2 in rural towns in New South Wales, Australia. Of the urban groups, 2 specifically targeted young people aged 18 to 28 years. A further 20 people were involved in the interviews, all of whom lived in Sydney, Australia.

Table 1 provides demographic and mental health data for the participants involved in the 3 related studies.

**Table 1:** Participants’ characteristics

		Online Survey (n = 525)		Focus Group Discussions (n = 47)		Interviews (n = 20)	
		%	n	%	n	%	n
**Gender**							
	Female	67.6	355	70	33	60	12
**Age category**							
	18-24	16.6	87	53	25	25	5
	25-34	31.8	167	13	6	20	4
	35-49	38.1	200	21	10	45	9
	50-59	10.3	54	6	3	5	1
	60+	3.2	17	6	3	5	1
**Employment status**							
	Employed full-time	43.6	229	14.9	7	50	10
	Employed part-time	14.9	78	17	8	5	1
	Self-employed	8.2	43	14.9	7	25	5
	Full-time student	13.3	70	38.3	18	10	2
	Unemployed	3.8	20	6.4	3	10	2
	Retired	1.7	9	6.4	3	0	0
	Home duties	6.7	35	2.1	1	0	0
	Temporarily unable to work due to illness or injury	6.1	32	0	0	0	0
	Permanently unable to work due to illness or injury	1.7	9	0	0	0	0
**Relationship status**							
	Single	37.7	198	66	31	35	7
	De facto relationship	16.6	87	6.4	3	15	3
	Married	36	189	23.4	11	30	6
	Divorced/separated	9.5	50	2.1	1	15	3
	Widowed	0.2	1	2.1	1	5	1
**English spoken at home**							
	Yes	92	483	93.6	44	75	16
**Mental health**							
	Current depression	60.2	316	23.4	11	30	6
	Current anxiety	49.1	258	19.1	9	25	5
	Current stress	51.8	272	19.1	9	45	9
	Current treatment for depression	54.3	285	23.4	11	10	2
	Current treatment for anxiety	30.5	160	10.6	5	20	4
	Current treatment for stress	17.9	94	6.4	3	5	1
	Lifetime depression	92	483	83	39	60	12
	Lifetime anxiety	65	341	46.8	22	25	5
	Lifetime stress	77.7	408	66	31	55	11

### Analysis

Data from each component of the study were analyzed separately and then triangulated. Triangulation involves the exploration of a research question by using multiple data gathering methods in order to get a better understanding of the subject matter, to cross-check the research findings, and to increase their validity [15]. In our studies, we also used the information from the focus group discussions and interviews to shed light on and illustrate the findings of the online survey. Descriptive analyses and tests of group differences within the survey data were conducted using PASW Statistics Version 18 (SPSS Inc, Chicago, IL, USA). Within the interviews and focus group transcripts, salient themes and principles were identified using the “thematic analysis” technique, a qualitative method for identifying, analyzing, and reporting patterns of meaning within data [16]. Data are organized and described in rich detail within a theoretical framework. In this study, an existentialist or realist framework was used whereby the experiences, meanings, and the reality of participants were identified and reported as expressed, in contrast to other frameworks which focus on, for example, the manner in which participants’ meanings are “constructed” within the broader context of society [16].

## Results

### Current Mobile Phone Behavior

**Figure 1:**
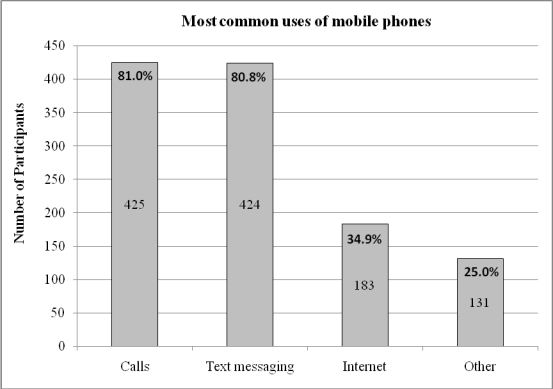
Most common uses of mobile phones by online survey respondents

**Figure 2:**
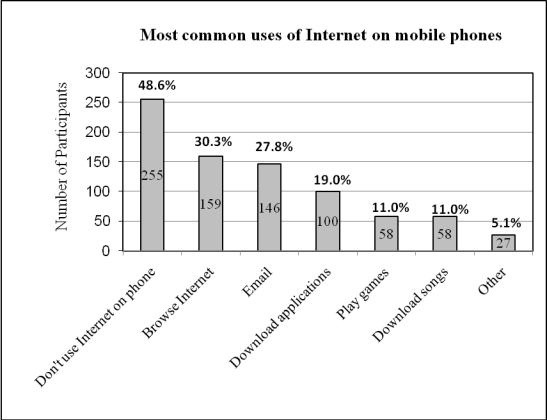
Most common uses of the Internet on mobile phones

The majority of survey respondents (455/525 or 86.7%) owned a mobile phone or personal digital assistant (PDA) of whom 83.3% (379/455) reported using it at least daily. Making and receiving calls and sending and receiving short message service (SMS) messages were the predominant functions used (see Figure 1). Other functions commonly used included the Internet (for downloading songs and videos, listening to music, accessing email and Facebook, and listening to the radio: see Figure 2), camera, alarms, memos and reminders, calendar and appointments, clock, games and calculator.

Nearly half (255/525 or 48.6%) indicated that they did not use their mobile phone to access the Internet. Reasons included the cost (150/525 or 28.5%), lack of need because they have Internet access on a computer (169/525 or 32%), their mobile phone is not Internet-enabled (66/525 or 12.6%), they haven’t had the need (67/525 or 12.7%), or a variety of other reasons, such as not knowing how to access the Internet via the mobile phone, finding it too difficult or complicated, or because the phone is poor quality with a small screen.

Focus group and interview findings converged with those of the online survey, with the exception of accessing the Internet. All focus group participants owned a mobile phone and the majority (39/47 or 83%) said they used it every day, predominantly for social reasons, but 72% (34/47) said they also used their mobile phone for work. However, only 18/47 (38%) accessed the Internet on their mobile phone and this was primarily for email, Facebook, Twitter, music, directions, games, Google, and Internet browsing. Reasons participants gave for not using the Internet on their mobile phone included the cost, not knowing how to use it, having no need to use it, or the Internet was not available on their mobile phone.

Responses from interviewees were similar. While all owned a mobile phone or PDA, usage varied widely from a few times a week to more than 50 times a day. However, similar to the survey respondents and the focus group participants, the majority (19 of the 20 interviewees) reported using their phone at least once a day. The different uses to which their mobile phones were put were also similar: all participants reported making phone calls, 18/20 (90%) used text messaging, and a minority used their mobile phone for checking emails, taking photos, and other functions such as a clock, a calculator, a calendar, or an alarm or to set reminders. However, 13/20 (65%) did not use the Internet on their phone, and a similar proportion reported that they did not know how to download a program or application on their phone. The reasons participants gave for not using the Internet on their mobile phone were that they had Internet access at home or work, the cost was too high, or their phone didn’t allow Internet access.

### Attitudes Toward Using a Mobile Phone for Mood Monitoring and Self-help

#### Interest in Using a Mobile Phone Program for Monitoring and Self-management

The majority (399/525 or 76%) of survey respondents indicated that they would be definitely be interested (245/525) or likely (154/525) to be interested in using a program on their mobile phone to monitor and manage their mood, anxiety, or health. Among the 455 respondents who owned a mobile phone or PDA, there were no significant differences between those who were interested in using their mobile phones in this way and those who were not by gender (χ2_1_, = 0.98, *P* = .32, phi = .05), age group (χ2_4_, = 1.95, *P* = .75, phi = .06), employment status (χ2_2_, = 2.74, *P* = .25, phi = .08) or marital status (χ2_4_, = 4.62, *P* = .33, phi = .10). However, the presence of symptoms of depression, anxiety, or stress was associated with reported increased interest in using such a program (χ^2^_1_, = 16.67, *P* < .001, phi = .19). Specifically, standardized residuals indicated that among participants who stated that they were not interested in using the program, there were fewer with current mental health symptoms than expected (and an overrepresentation of participants with no symptoms; *P* < .05 and *P* < .01, respectively). Of the 9.9% (45/455) of respondents who reported current symptoms of depression, anxiety, or stress and were not interested in the program, 68.9% (31/45) indicated that they did not think using a mobile phone program to track moods could help people to manage their depression, anxiety, or stress.

These results were supported by information from the focus group discussions. The majority (33/47 or 70%) of focus group participants also said they were interested in the notion of using their mobile phone to track their mood, anxiety, or health. Reasons given included speed, convenience, ease of access, the importance of being able to monitor and reflect on mood changes during the day, the opportunity to improve self-awareness, self-management, and well-being, access to support when it was not possible to get to a doctor, the view that it would be less confronting than face-to-face consultation, and the possibility of helping isolated people feel connected. Comments included:

A mobile phone application would be a highly convenient, portable, and discreet way of tackling one’s condition.

Everyone uses the Internet and mobile phones.

It could help those who are isolated and have mental health issues.

You have your phone with you most of the time, so you would be able to record moods more accurately.

Reasons given by those who were not interested in using their mobile phone to monitor their mental health included not liking to use their phone or technology, concerns that it would be too intrusive or privacy would be lacking, and not seeing the benefit of tracking mood and behavior. For example:

If the technology is too difficult, then it only adds to the stress and a sense of failure.

It seems impersonal and too generalized to be able to capture the emotions of each individual.

I can’t see the connection between tracking mood and lowering anxiety, stress, [or] depression.

#### Mode of Using the Program

Of the 399 survey respondents who indicated they were interested in using a monitoring and management program delivered via their mobile phone, 93.7% (374) indicated that they would want a username and password to log on. The length of time per session for which they would be prepared to use such a program ranged from 1 to 90 minutes (median = 5 minutes). The mean number of mood dimensions that participants were interested in tracking at any one time was 5.6 (SD 3.2). Most of these respondents (329/399 or 82%) thought that they would use the program at least daily, some suggesting multiple times per day (196/399 or 49.1%). However, there were significant differences in the expected mode of use according to whether respondents reported current symptoms or not. The participants who were without current symptoms (96 out of 399) indicated that they would use such a program less often (χ2_1_, = 4.52, *P* = .03, phi = .11) and for shorter periods (mean 7.28 minutes, SD 6.03) than the 303 respondents with current symptoms (mean 10.7 minutes, SD 11.28; *t*_301_ = 3.82, *P* < .001 [two-tailed]).

Information presented in the interviews illustrated the online survey findings. For example, comments from the 13 (of 20) interviewees who reported that a program to monitor depression, anxiety, or stress would definitely be helpful included:

It is text savvy and good for the younger generation.

Yes, it would be great, another avenue to use and to help people feel that they are not alone.

It could be a great motivational tool for people to look after themselves better.

Maybe [it would be good] if someone cannot afford psychological treatments or for people who like to keep their feelings to themselves.

In addition, 5 interviewees felt that such a program might be helpful, but with caveats. For example, one asked, “If someone such as a doctor is already helping to manage one’s moods, why would you use the Internet?” Another 2 interviewees thought that face-to-face contact with a professional would be more beneficial.

In response to the question of whether they would use the program themselves if there were no cost, 17 answered positively, and the majority said that they would use it at least once a day.

I would be very interested [in using the program] because it’s a new tool and not intrusive, and it would be handy.

I would be interested, not for my moods but for my health.

Other reasons given included “my phone is always with me.” 

Of the interviewees who reported that they would not use a mobile phone mental health program, the reasons given included that they use their mobile phone only for basic functions like phone calls and they did not see how monitoring moods or behavior would help them if they were depressed, stressed, or anxious.

#### Key Functions and Features Required

**Figure 3:**
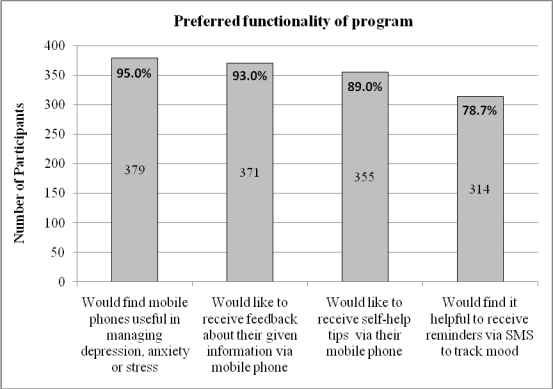
Preferred functionality of program: online survey respondents

In answer to questions about the key functions required in such a program, 78.7% (314/399) of the survey respondents interested in using the program said they would find it helpful to receive SMS reminders to track their moods; 93% (371/399) nominated that they would want to receive feedback about the information they had entered into such a program; and 89% (355/399) were interested in receiving self-help suggestions (Figure 3). Comparison of respondents with (303/399) and without (96/399) current depression, anxiety, or stress showed that significantly more of those with symptoms indicated that they would find it beneficial to receive SMS reminders to track their moods and behaviors (χ2_1_, = 9.98, *P* = .002, phi = .165). However, there was no significant difference between those with and without current symptoms in whether they saw feedback on monitoring information as a requirement (χ2_1_, = .01, *P* = .91, phi = -.02) or whether they would want to receive self-help suggestions (χ2_1_, = .51, *P* = .47, phi = -.04).

**Figure 4:**
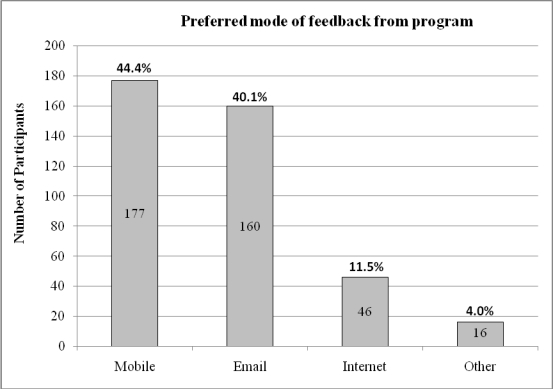
Most preferred modes of feedback from program among online survey respondents

**Figure 5:**
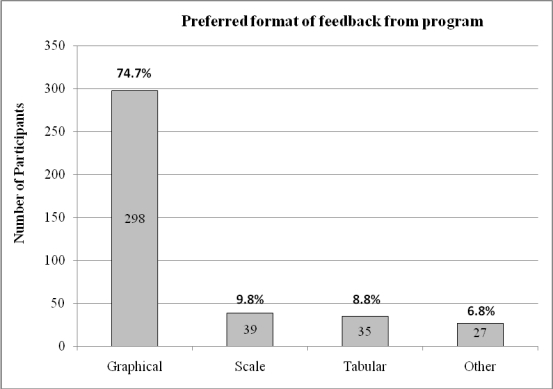
Most preferred formats of feedback among online survey respondents

As can be seen in Figure 4, choice of delivery channel to receive the feedback and self-help suggestions was mixed: 44.4% (177/399) opted for their mobile phone, 11.5% (46/399) chose the Internet, and 40.1% (160/399) chose email. Figure 5 illustrates that the preferred format for such feedback was graphs (298/399 or 74.7%) rather than tables or scales. In answer to the question of whether they would be more inclined to use the program if it had games or fun activities, nearly two-thirds (257/399 or 64.4%) of survey respondents interested in using a mobile phone mental health program said no. However, a similar proportion (250/399 or 62.7%) said they would want the option of personalizing the program with colors, background, logos, and so on.

Detailed discussion took place in the focus groups about what constituted necessary features of a mobile phone mental health program. Privacy was highlighted as an issue of significant importance and the majority said that a secure log-in comprising username and password should be a mandatory feature. The need for the program to be simple to use and “foolproof” was also emphasized. Usage should be quick (maximum 10 minutes) and easy; one participant suggested that the benchmark for its ease and privacy of use was whether it could be used on public transport. SMS reminders were seen as helpful as long as users had the option of varying their delivery so that they did not become intrusive. Feedback was deemed to be very important, and data presented in graphical form was the most popular format suggested. Functionality allowing day-to-day and week-to-week comparison was also seen as important. Participants suggested that entering data on the mobile phone and receiving it via computer would be an acceptable method that would also resolve some issues of privacy. Allowing users flexibility of choice regarding how much information is to be fed back and over what time frame was also seen as key.

Interviewees reported similar attitudes. They highlighted the importance of privacy and security of information, and the majority said they would want a username and password to access the program. A majority also indicated that they would want to receive short message service (SMS) reminders to track their moods/behaviors. The minority who did not want reminders indicated that they would find them annoying and bothersome.

If given the choice, 86.2% (344/399) of survey respondents interested in using a mobile phone mental health program indicated that they would allow their doctor to access or receive information about their moods and behavior from the program. Interviewees were similarly minded, saying that “it would be good to have a human on the end to make sense of the data I enter,” and “yes, it would be a helpful, precise, and efficient way of tracking what’s happening.” Of the 91 survey respondents who were against the idea, the major reason given was that it would constitute a breach of privacy for them, while others explained that they don’t have a doctor whom they see regularly. There was an even split between those with current symptoms (48/91 or 52.7%) and those without. One interviewee said he did not see the point because “if I am seeing a doctor for my health or mental illness, I would already be telling him what is going on.”

## Discussion

This study explored, for the first time, community attitudes toward the appropriation of mobile phones for mental health monitoring and self-help. Triangulation of results from the 3 study components suggests that, overall, participants were positive about the idea of conceptualizing mobile phones as a mental health tool but the acceptance was conditional upon a number of key features being included. These included the need for the program to be simple and straightforward to use and the need for its security and privacy to be guaranteed, especially for information sent to the mobile phone. A user name and password were considered to be mandatory. Text message reminders were seen as helpful as long as they were not intrusive, and feedback graphs were deemed to be important.

However, there were differences in expected mode of use between participants with current depression, anxiety, or stress and those without. Respondents with current symptoms indicated that they would be prepared to use a mobile phone program more often and for longer periods, and they were also significantly more likely to want to receive SMS reminders to track their moods and behaviors. Nevertheless, both groups indicated that they would want feedback on monitoring information and to receive self-help suggestions from such a mobile phone program.

Thus, while there appears to be a community willingness to accept a broadening of the conceptualization of mobile phones to embrace functions associated with improving mental health, there are caveats to the appropriation of mobile phones for the new functions. The implications for clinicians and eHealth providers are clear. Most mental health programs, whether delivered face-to-face, by telephone, book, or computer, rely on their clients monitoring their symptoms or activities, either as an integral component of the service or as a complement to it. The information is useful for individuals to help them gain control of their condition and for service providers to review the effectiveness of their service. However, to successfully facilitate self-monitoring and self-management via mobile phones, clinicians and eHealth developers must place additional importance on ensuring that the mobile phone programs are secure, private, and easy to use.

A further implication arising from the data concerned the apparent lack of understanding about the rationale for and benefits of mood tracking among some respondents. Nearly 10% of the sample, the majority of who reported current depression, anxiety, or stress, indicated that they did not see how a mobile phone program for monitoring moods would help people manage their depression, anxiety, and stress. It is not known if they were expressing doubt about monitoring per se or monitoring specifically on a mobile phone. If the former, then considering that the recent National Survey of Mental Health and Wellbeing in Australia [17] found that 65% of people with mental health conditions do not access services, our results suggest that a health promotion campaign outlining the benefits of self-monitoring for mental health may be helpful.

### Limitations

Having been recruited primarily through the websites of the University of New South Wales and the Black Dog Institute, the intranets of a variety of companies and consumer organizations, and via Facebook, the convenience sample for the online survey is likely to be unrepresentative of the broad population. Visitors to the site were self-selected, and because Internet access is not equal among all socioeconomic and demographic groups, biased estimates on variables related to socioeconomic status may have resulted [18]. Nevertheless, we felt it was justified to recruit from these online sources because our research questions pertained to the use of electronic technologies. Post hoc inspection of the survey sample indicates that ownership and usage of mobile phones was representative of the Australian population. However, there was a stronger representation of survey respondents with current or lifetime depression, anxiety, or stress compared with the Australian population. Another limitation was that our studies took place only in Australia and, although the prevalence of mental health problems in Australia is similar to that in other developed countries and mobile phone penetration is as high, the results may not generalize to populations in other developed countries.

### The Future

In line with the results of our research, we are now developing a mobile phone monitoring and self-help program at the Black Dog Institute. The “myCompass” system will provide users with a tool to monitor and manage depression, anxiety, and/or stress via the Internet on their mobile phone or computer. With the assistance of optional screening questions, users can receive tailored suggestions about mood and behavior dimensions they might find helpful to monitor, or they can select from a menu of monitoring dimensions themselves. They can also choose the time of day they want to monitor and whether they would like to receive regular SMS or email messages to prompt them. Both real time and retrospective assessment of moods, events, and behavior will be available, and users will be able to receive graphical feedback of their data with situational information if desired. They may also choose to receive brief self-management modules (involving interactive cognitive-behavioral strategies), motivational messages, stories, information, and tips to help them to manage depression, anxiety, and stress. Alternatively, the system will offer a selection of strategies derived from algorithms based on user data. The program is designed as a stand-alone tool for the public that is secure and easy to use.

Once the digital program is fully developed and pilot tested, a large-scale randomized controlled trial is planned to measure outcomes arising from use of the program.

### Conclusion

Mobile phones are the way of the future. Ownership has reached saturation point in most developed countries, and in developing countries it is increasing exponentially. The simplification and rapid development of digital technology together with the way in which mobile phones are carried on the person and switched on and positive community attitudes make the mobile phone a useful vehicle for enhancing access to evidence-based monitoring and self-help for people with mild to moderate high-prevalence mental health conditions.

## Multimedia Appendix

Advertisement for the online survey

## Abbreviations

EMA: Ecological Momentary Assessment
IP: Internet protocol
PDA: personal digital assistant
SMS: short message service
