# Supplementary material for: Community Attitudes to the Appropriation of Mobile Phones for Monitoring and Managing Depression, Anxiety, and Stress
Source: J Med Internet Res. 2010 Dec 19;12(5):e64. doi: 10.2196/jmir.1475 (PMC3057321; doi:10.2196/jmir.1475)
Supplement: Supplementary file 1 [file jmir_v12i5e64_app1.pdf]

Multimedia Appendix 1. Advertisement for the online survey

THE UNIVERSITY OF NEW SOUTH WALES SYDNEY · 2052 · AUSTRALIA	BLACK DOG INSTITUTE

MOBILE PHONES as a HEALTH TOOL?

WE WOULD LOVE **YOUR** VIEWS!!

We are looking for volunteers to complete a 15 minute online survey. The survey is about the use of mobile phones to help people track their moods and behaviour and learn how they can manage depression, anxiety and stress.

We are interested in hearing the views of people with and without depression, anxiety or stress.

The questionnaire is completely confidential.

Everyone who completes the survey can enter the draw for an 8GB IPOD Nano.

Can I do the survey?
If you are 18 years or older we would like to invite you to complete the survey.

What do I need to do?
If you are interested in filling out the survey, please follow the link to the survey.

http://www.blackdoginstitute.org.au/docs/MobileTrackerSurvey2.pdf

Further information is available the website above or can be gained by contacting XXXXX, email: xxxxx
